# Mechanisms of unconventional CD8 Tc2 lymphocyte induction in allergic contact dermatitis: Role of H_3_/H_4_ histamine receptors

**DOI:** 10.3389/fimmu.2022.999852

**Published:** 2022-10-07

**Authors:** Julieta Alcain, Alejandra del Pilar Infante Cruz, Gabriela Barrientos, Silvia Vanzulli, Gabriela Salamone, Mónica Vermeulen

**Affiliations:** ^1^ Instituto de Medicina Experimental (IMEX), CONICET, Academia Nacional de Medicina, Buenos Aires, Argentina; ^2^ Laboratorio de Medicina Experimental, Hospital Alemán, Buenos Aires, Argentina; ^3^ Laboratorio de Anatomía Patológica, Instituto de Estudios Oncológicos, Academia Nacional de Medicina, Buenos Aires, Argentina; ^4^ Departamento de Microbiología, Parasitología e Inmunología, Facultad de Medicina, Universidad de Buenos Aires, Buenos Aires, Argentina

**Keywords:** H_4_/H_3_ histamine receptors, allergy contact dermatitis, non-conventional CD8^+^ lymphocytes, immunoregulation, dendritic cells

## Abstract

Histamine (HA) is a potent mediator that plays a central role in inflammation and allergy, acting through four G-protein-coupled receptors (i.e. H_1_–H_4_). HA is an accepted promoter of type 2 immunity in CD4^+^ T cells during hypersensitivity. Previously, we demonstrated that HA can promote antigen cross-presentation, inducing the activation of antigen-specific CD8^+^ T cells in an asthmatic murine model. Non-classical CD8+ T-cell profiles, such as Tc2 or Tc17, are associated with allergic disease persistence and chronicity. In this paper, we focus on the role of the H_3_ receptor (H_3_R) and the H_4_ receptor (H_4_R) in the development of allergic contact dermatitis. We were able to show that induction of the type 2 profiles associated with interleukin 13 production, both by CD4 and CD8 lymphocytes, depend on the interaction of HA with H_3_R and H_4_R. Blocking both receptors using the selective H_3_/H_4_ receptor antagonist thioperamide or the selective H_4_R ligand JNJ777120 reduces the inflammatory response, inducing an immunosuppressive profile associated with the increased proportion of FOXp3^+^ regulatory T lymphocytes and CD11b^+^Gr-1^+^ myeloid suppressor cells. Interestingly, in dendritic cells, only H_4_R blockade, and not H_3_R blockade, is capable of modulating most of the inflammatory effects observed in our model.

## Introduction

Contact dermatitis (CD) can be defined as the formation of erythema and eczema in the skin after a single application of a sensitizing agent. Skin damage is associated with the accumulation of Th1/Tc1 or Th17 lymphocytes at the site of inoculation (1, 2). Interestingly, repeated application of the sensitizing agent induces a change from Th1 to Th2/Tc2 in the epidermal microenvironment, resulting in chronic contact allergy, i.e., allergic contact dermatitis (ACD) (3). Haptens (4) are known sensitizers in CD. Haptens must be associated with epidermal proteins to induce the adaptive response. 2,4-dinitro-fluorobenzene (DNFB) is an irritant currently used in mice as a classical model of ACD. Characteristically, Langerhans cells internalize the hapten–protein complex, starting their migration at the lymph node to initiate an adaptive response. The effector response is associated with the induction of a T-helper immune response (5–7). It should be noted that non-conventional CD8 lymphocytes can be categorized into different subtypes called Tc2, Tc17, and Tc9 depending on the profile of cytokines secreted (Th2, Th17, or Th9, respectively). In particular, CD8^+^ Tc2 lymphocytes are observed in allergic disorders, and their abundance is correlated with disease severity (8).

Histamine (HA), as a biogenic amine, is associated with the development of allergic and inflammatory responses through its known effect on smooth muscle contraction and bronchospasm (9, 10). The physiological functions of HA are mediated through four HA receptors, H_1_R, H_2_R, H_3_R, and H_4_R. For years, inflammatory diseases triggered by HA were thought to be caused by their interaction with H_1_R (11, 12). H_1_R antagonists are known to have beneficial effects in some conditions, such as pruritus and rhinitis. However, in some cases, unwanted effects have been observed, and H_1_R antagonists were unable to inhibit asthma symptoms (13–15). Since the identification of H_4_R antagonists (16, 17), this group of drugs has been evaluated as potential targets of inflammatory effects of HA in several pathologies affecting the skin and mucosal tissues. In a murine model of Th2 inflammation, several authors demonstrated that H_4_R signaling mediates inflammation and pruritus (18). Later, however, it was shown that pruritus sensation is not associated with H_4_R (19), but that the role of H_4_R in inflammation is related to the expression of H_4_R on T lymphocytes, and that upregulation of H_4_R leads to increased expression of interleukin 4 (IL-4) (19–21). As single HA receptor antagonist therapies are not particularly effective in minimizing symptoms of allergic reactions, combination therapies have recently been evaluated (22, 23) for their ability to alleviate symptoms. It was found that a combination of H_1_R and H_4_R antagonists had desirable effects, such as inhibition of the Th2 profile, a reduction in IgE, and improvement in eczema (24). Tamaka et al. (25), in a model of chronic CD induced by tetrachloride-nitrobenzene, demonstrated that HA induced the recruitment of Th2 cells in the skin, and resulted in an increase in IgE levels in the serum, of sensitized mice, effects that were diminished in the presence of H_1_ or H_4_ antagonists. Studies evaluating the role of HA antagonists in allergic dermatitis have revealed substantial differences in their protective role, particularly in the case of H_4_R antagonists, and these differences may be attributable to the sensitizers used. In addition, in general, the effects observed were more pronounced in mice in which the H_4_R gene was knocked out (i.e., H_4_R^–/–^ mice) (26, 27) than in normal mice in which the antagonist was applied topically. For example, in H_4_R^–/–^ mice, the induction of skin lesions by ovalbumin is inhibited (17), but the application of H_4_R antagonists is not effective in the same model, probably because local concentrations are insufficient. In this work, we analyze whether HA induces the production of unconventional cells in a model of allergic CD and the mechanisms underlying this effect. We found that H_4_R blockade or dual H_3_R/H_4_R blockade through the use of locally administered antagonists is associated with the reversal of the hapten-induced effect and that this is due to the generation of a tolerogenic microenvironment as a result of increased production of regulatory T cells and IL-10.

An important finding is that H_4_R blockade in dendritic cells (DCs) reproduced the effects observed by topical JNJ7777120; however, this modulation was not observed when using the dual antagonist thioperamide.

## Materials and methods

### Mice

All experiments were carried out using 2-month-old virgin female BALB/c mice, which were raised at the National Academy of Medicine, Buenos Aires, Argentina. The mice were housed three or four per cage, assigned to each treatment, and kept at 20 ± 2°C under an automatic 12 hours/12 hours light–dark schedule. Animal care was in accordance with institutional guidelines. All experimental protocols were approved by the Institutional Animal Care and Use of the Experimentation Animals Committee (CICUAL number 2015/02).

### Contact dermatitis

Animals were sensitized for 5 days by topical administration of 20 µl of DNFB 0.3% diluted in acetone/water (in a ratio of 3:1). DNFB was applied to the medial inner flank of the ears daily for 5 days. Some groups also received, together with DNFB, the of H_3_R/H_4_R dual antagonist thioperamide (1 µM/day) or the selective H_4_R antagonist JNJ7777120 (JNJ) (1 µM/day). On day 7, the effector phase was elicited by topical application of 20 µl of DNFB dissolved in phosphate-buffered saline (PBS). One day later, tissue induration was measured with a caliper, the animals were euthanized by cervical dislocation, and blood, draining lymph nodes, and injured tissue were recovered.

### Immunochemistry

Isolated ear tissue sections were fixed in 4% paraformaldehyde in PBS for 24 hours and then embedded in paraffin. Four-micron sections were stained with hematoxylin and eosin, and Giemsa. Representative microphotographs were taken with a Ziess Axiolab microscope (Zeiss, Oberkochen, Germany).

### Dendritic cells generation from bone marrow cultures

To obtain murine DCs, bone marrow was flushed from the limbs of long bones using 2 ml of RPMI-1640 (Invitrogen, Carlsbad, CA, USA), a syringe, and a 25-gauge needle. Red cells were lysed with 0.45 M ammonium chloride in PBS (1 ml) for 1 minute, and then the reaction was stopped by the addition of 30 ml of RPMI-1640. After washing, cells were suspended at a concentration of 1.5 × 10^6^ cells/ml in a solution comprising 70% complete medium [Gibco RPMI-1640 medium supplemented with 10% fetal bovine serum (FBS) and 5.5 × 10^–5^ M mercaptoethanol (Sigma-Aldrich, St. Louis, MO, USA)] and 30% J588-GM cell line supernatant. The cultures were fed every 2 days by gently swirling the plates, aspirating 50% of the medium, and adding back fresh medium containing J588-GM cell line supernatant. On day 9 of culture, more than 90% of the harvested cells expressed MHC class II, CD40, and CD11c, but not Gr-1 (not shown). In some cases, lipopolysaccharide from *Escherichia coli* 0111:B4 at a concentration of 0.1 ng/ml was used as a control of DC activation.

### Preparation of dendritic cells to induce the effector phase in the dermatitis model

DCs obtained from bone marrow were adjusted to a concentration of 10^6^ cells/ml. Cells were then seeded in 96-well plates and treated with JNJ (1 µM) or thioperamide (1 µM) for 20 minutes at 37°C. The cells were then treated with HA (0.1 µM) for 30 minutes at 37°C. Finally, the DNFB hapten (100 µg/ml in 20% FBS) was incorporated and the cells were incubated for 1 hour at 37°C. After washing, 100 µl of cell suspension, containing 5 × 10^5^ DCs, was inoculated subcutaneously in the dorsal area near the ear. After 10 minutes, 20 µl of the hapten DNFB 0.3% diluted in acetone/water was topically applied. Topical administration of the hapten was carried out for a further 4 days.

### Cell disaggregation and flow cytometry

The cervical lymph node was removed, placed in RPMI-1640, and passed through a sterile mesh to obtain a single-cell suspension of mononuclear cells. The cells were collected in conical tubes and centrifuged at 1500 rpm/4°C. Mononuclear cells were then resuspended in a complete medium and cultured at 37°C or prepared for staining. The following monoclonal antibodies were used, conjugated with fluorescein isothiocyanate (FITC), phycoerythrin (PE), or peridinin chlorophyll (PerCP): anti-CD11c, anti-H2Kb, anti-CD11b, anti-GR1, anti-CCR7, anti-CD8, anti-CD4 (BD Pharmingen, San Diego, CA, USA). Data were collected using a FACSCalibur™ flow cytometer (BD Pharmingen) and were analyzed with Cellquest software (Becton Dickinson, San Jose, CA, USA). To perform intracellular staining, cells were fixed in 0.5% paraformaldehyde and permeabilized with saponin (0.1% in PBS). Permeabilized cells were incubated with PE-conjugated anti-mouse antibodies to IL-13 or IL-10 (BD Pharmingen), or with isotype-matched control antibodies, for 30 minutes. Finally, cells were washed twice with saponin buffer, suspended in Isoflow™ (BD Pharmingen), and analyzed by flow cytometry. In most cases, mononuclear cells were permeabilized using the eBioscience™ FOXp3/Transcription Factor Staining Buffer set (cat. no. 00-5523-00, ThermoFisher Scientific, Inc., Waltham, MA, USA) to identify anti-CD25, anti-CD4 and anti-CD8 antibodies (the characteristics of the antibodies used are in the **Supplementary Table S1**).

### Dendritic cell migration by FITC

Mice ears were treated topically with 1% FITC in PBS, with or without thioperamide (1 µM) or JNJ (1 µM). After 24 hours, the percentages of CD11c^+^FITC^+^ in suspensions obtained from zonal lymph nodes were analyzed by cytometry, as was described in “Cell disaggregation and flow cytometry.”

### Isolation of dendritic cells

To isolate DCs, cell suspensions obtained by passing cervical lymph nodes through a sterile mesh were centrifuged at 1500 rpm/4°C, as previously described. Cells were suspended in MACS buffer [PBS + 0.5% bovine serum albumin (BSA) and 2 mM EDTA pH 7.2] and, finally, DCs were purified by positive selection with the anti-CD11c Microbeat isolation kit (Miltenyi Biotec, Buenos Aires, Argentina) (purity obtained = 95%). Magnetic separation was performed in accordance with the manufacturer’s instructions.

### Proliferation assay

The proliferation assay was measured by carboxyfluorescein succinimidyl ester (CFSE) dilution. Mononuclear cells were labeled with the fluorescent dye CFSE (5 µM) and seeded in 96-well plates containing anti-CD3 (0.1 µg/ml) at a concentration of 2.5 × 10^5^ cells/well. After 96 hours, cells were recovered and CFSE fluorescence was determined by flow cytometry. Proliferating cells are those displaying a lower intensity of fluorescence because CFSE is divided between daughter cells.

### Analysis of serum levels of IgE and IgG antibodies directed to DNFB

At the end of the experiments, serum samples were obtained from mice by cardiac puncture. DNFB-specific IgE and IgG antibodies were detected using coated plates incubated overnight with 1 μg/ml DNFB in sodium carbonate buffer (pH 9.5; Sigma-Aldrich). Plates were treated with Tween 0.5% in PBS (TPBS), supplemented with 1% BSA, for 2 hours at room temperature. Serial dilutions of sera were added and, after 2 hours, the plates were washed three times with TPBS and an appropriate dilution of the biotinylated detection antibody (i.e., rat anti-mouse IgE or IgG; BD Pharmingen) was added for 1 hour. After the plates had been washed, the enzyme avidin peroxidase (eBiosciences, San Diego, CA, USA) was added for 20 minutes. 3,3′,5,5′-tetramethylbenzidine was used as a substrate. Absorbance was measured at 450 nm.

### Cytokine determination

The cytokine levels in supernatants of mononuclear cells were measured by ELISA. Assays for interferon gamma (IFN-γ) and IL-13 (eBiosciences, San Diego, CA, USA) were performed in accordance with the manufacturer’s protocols.

### RNA extraction and reverse transcription

Total RNA was extracted from the ear using TRIzol reagents (Gibco-Life Technologies, ThermoFisher Scientific Inc.). The reverse transcription reaction contained 1 μg of RNA and was performed using Moloney murine leukemia virus reverse transcriptase (Promega, Madison, WI, USA). The blastx (National Center for Biotechnology Information, Bethesda, MD, USA), Primer3Plus (Whitehead Institute for Biomedical Research, Cambridge, MA, USA), and Beacon Designer (Premier Biosoft, Palo Alto, CA, USA) programs were used for the design of primers specific for the desired sequences. They were analyzed for optimal annealing and melting temperatures, the optimal amount of cytosine and guanine bases, and minimal formation of secondary structures.

### Quantitative PCR

The DNA copy was amplified using specific primers designed for the sequences of the following cDNAs: IL-17, IL-5, IL-13, and IL-10 (the specifications of the sequences are detailed in **Supplementary Table S2** *)*. Amplification was performed using a SybrGreen Master Mix (BioRad Laboratories, Inc., Hercules, CA, USA) and a CFX-Connect thermocycler (BioRad Laboratories, Inc.). The program used for the analysis was CFX Maestro (BioRad Laboratories, Inc., Hercules, CA, USA). The ΔCt of each gene compared with the control gene (i.e., glyceraldehyde 3-phosphate dehydrogenase) is reported for each of the treatments.

### Statistical analysis

Graphs were created using FlowJo X (FlowJo, LLC, Ashland, OR, USA) and GraphPad Prism 6 (GraphPad Software Inc., CA, USA). The statistical analyses used were ANOVA with Tukey’s post-test and, to determine differences between the two means, the Student’s *t*-test. In all cases, a *p*-value of < 0.05 was considered statistically significant.

## Results

### Blockade of H_3_R and/or H_4_R inhibit inflammation in a dermatitis model

**Figure 1:**
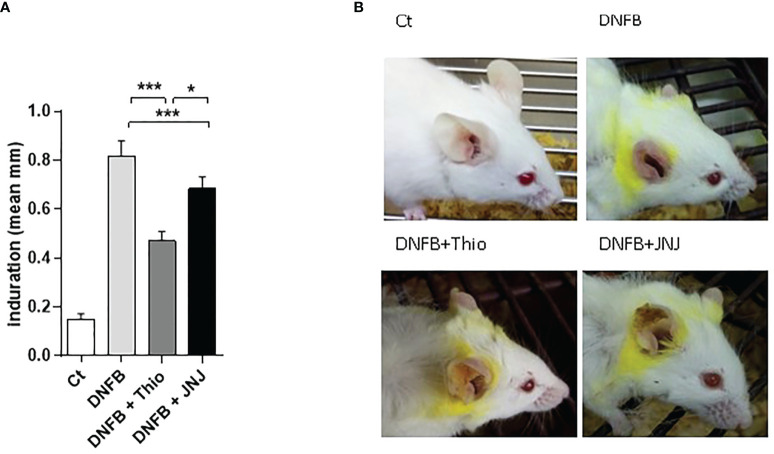
**(A)** Blockade of H_3_R and H_4_R decreases hapten-induced local inflammation in the ear (determined as ear thickness, measured with a caliper). The bars represent mean ± SEM values from nine mice. *p < 0.05; ***p < 0.001. ANOVA with Tukey’s post-test (*n* = 9). **(B)** A representative image of the macroscopic appearance of the ear tissue at day 7 post challenge, for each treatment.

**Figure 2:**
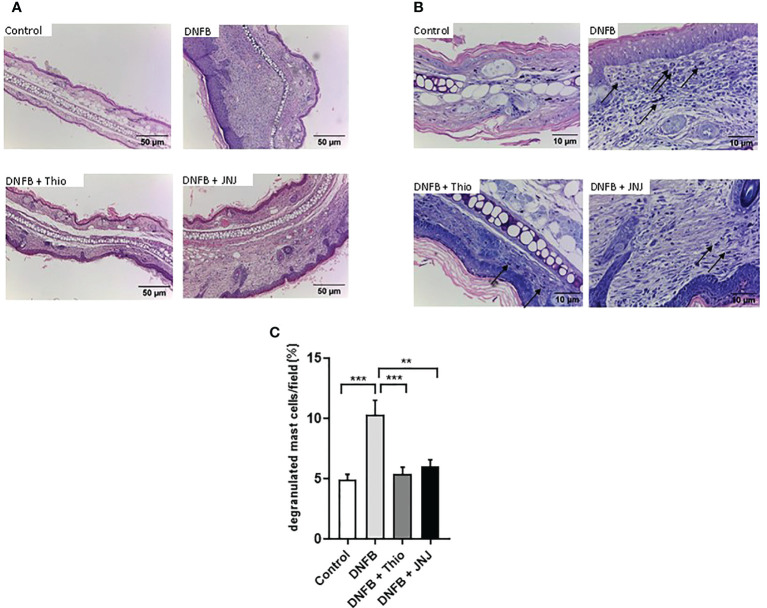
**(A)** Microscopic analysis of topical tissue stained with hematoxylin and eosin (×10 magnification). **(B)** Degranulated mast cells stained with Giemsa are indicated by arrows (×40 magnification). Representative micrographs of three independent experiments are shown. **(C)** Degranulated mast cells in tissue sections of the ear were counted in four fields chosen at random. Data are presented as the mean ± SEM of three independent experiments using three mice. **p < 0.005; ***p < 0.001.

As HA is a known modulator of inflammatory symptoms associated with allergic dermatitis (9, 28), we evaluated the role of H_3_R/H_4_R and H_4_R antagonists in the inflammatory response triggered by the DNFB. **Figure 1A** shows induration, measured as the thickness of the ear. Induration is significantly reduced by the use of thioperamide and JNJ antagonists, indicating that both receptors are involved in generating the damage in this model. As shown in representative tissue images (**Figure 1B**), induration is accompanied by redness and significant tissue damage as a consequence of tissue edema, effects that are partly prevented by the use of both antagonists. Histological analysis of the ear sections revealed a large inflammatory mononuclear cell infiltrate in DNFB-treated mice (**Figure 2A**), as well as a large number of degranulated mast cells (arrows), which decreased in topical mice treated with thioperamide or JNJ (**Figure 2B**). Similarly, the quantification of degranulated mast cells in different randomly chosen areas of the tissue showed that both antagonists, compared with DNFB, reduced the number of mast cells by approximately 50% (**Figure 2C**).

**Figure 3:**
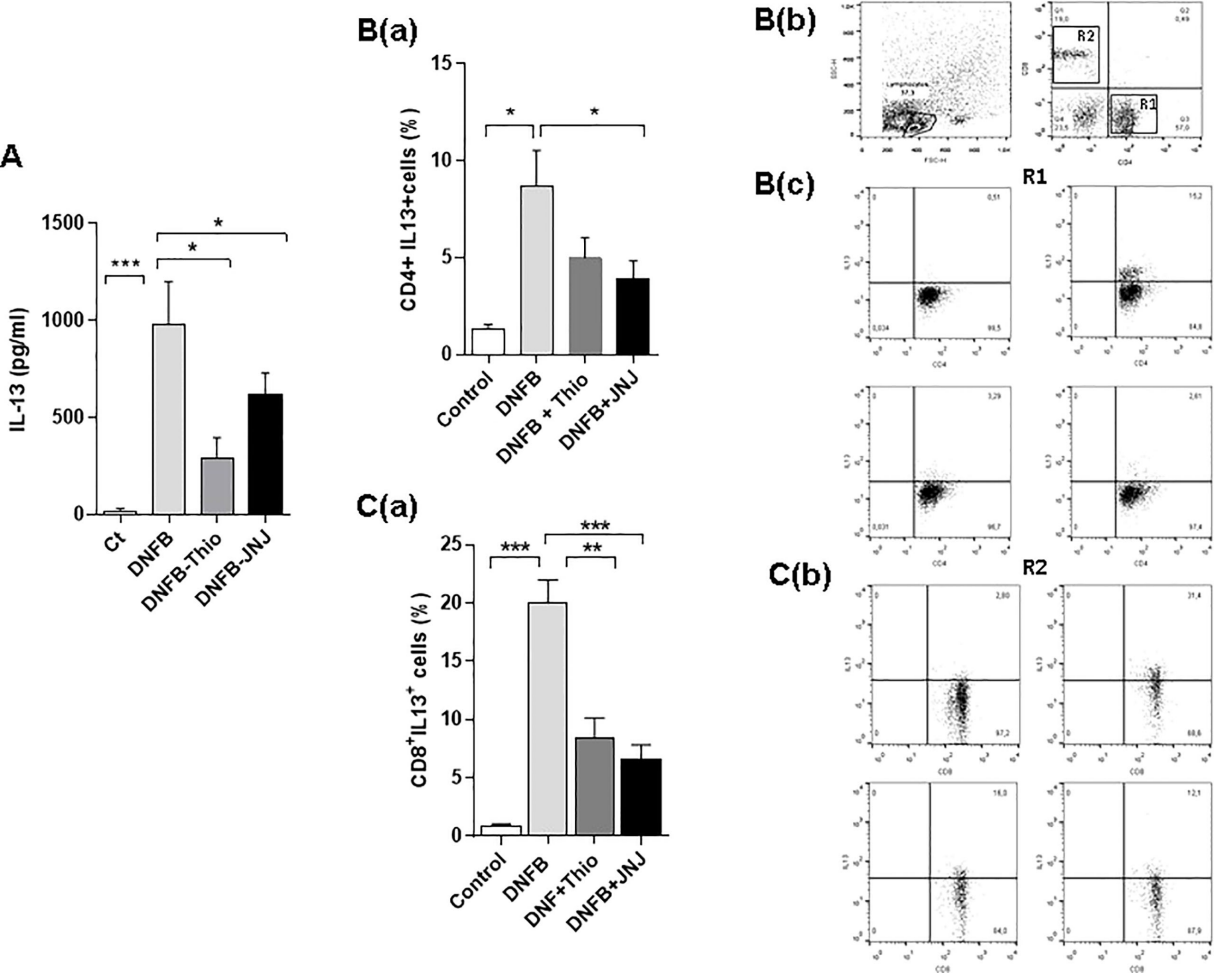
Blockade of H_3_R and H_4_R decreases the production of IL-13 in T lymphocytes. The determinations were made after 24 hours of stimulation of the cells with PMA (10 ng/ml) + ionomycin (1 ng/ml). **(A)** Quantification by ELISA in culture supernatants is shown. *p < 0.05; ***p>0.001. ANOVA with Tukey’s post-test (*n* = 7). The content of IL-13 in R1, corresponding to CD4^+^ cells (**B**, panel a), and in R2, corresponding to CD8^+^ (**C**, panel a) lymphocytes, is shown. The intracytoplasmic marking and flow cytometry, in the presence of brefeldin (10 µg/ml). The cells were permeabilized and labeled with PE-labeled IL-13 antibody. Bars represent the mean + SEM. *p < 0.05; **p>0.01; ***p < 0.001. ANOVA with Tukey’s post-test (*n* = 9). A representative fluorescence dot plot of CD4^+^ (**B**, panel c) and CD8^+^ (**C**, panel b) lymphocytes is shown.

**Figure 4:**
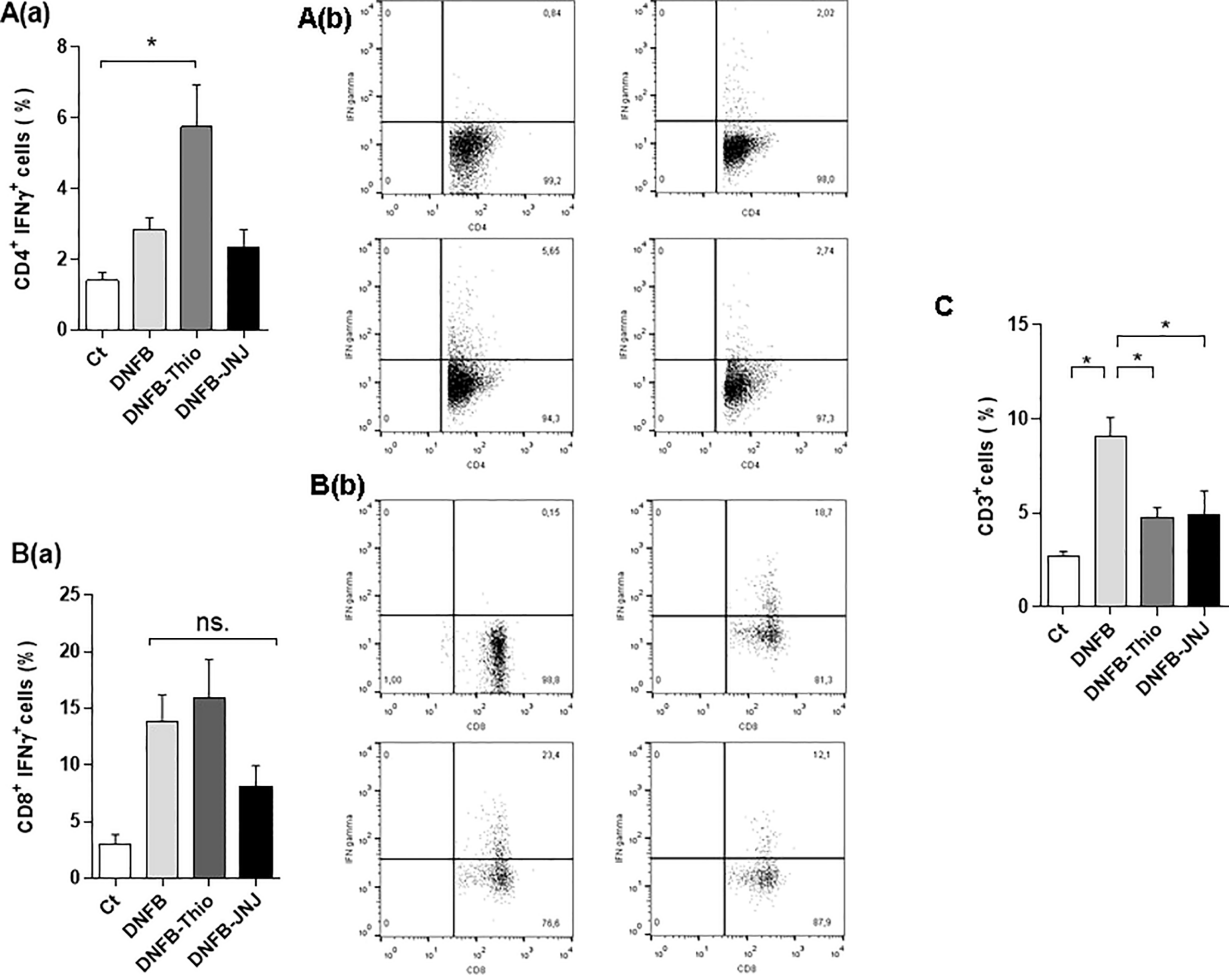
Analysis of the induction of a Th1/Tc1 profile. The bars represent mean ± SEM IFN-γ production by CD4^+^ (**A**, panel a) and CD8^+^ (**B**, panel a) cells in the gate of CD4 and CD8 lymphocytes, respectively. The intracytoplasmic determination was made from leukocytes from draining lymph nodes of treated mice after being cultured for 24 hours with PMA (10 ng/ml) + ionomycin (1 ng/ml), with the addition of 10 µg/ml brefeldin A (Golgi plug). Permeabilized cells were stained with PE-labeled IFN-γ. *p < 0.05. ANOVA with Tukey’s post-test (*n* = 8). A representative dot plot of CD4^+^IFN-γ^+^ (**A**, panel b) and CD8^+^IFN-γ^+^ (**B**, panel b) cells is shown. **(C)** The percentage of T lymphocytes (CD3^+^) at the administration site. Bars represent the mean ± SEM. ns (not significant); *p < 0.05. ANOVA with Tukey’s post-test (*n* = 9).

The analysis of cytokines showed that DNFB treatment increased the production of IL-13 in supernatants of leukocytes from lymph nodes, an effect that was reversed by treatment with both antagonists [i.e. thioperamide (1 µM/day) and JNJ (1 µM/day)] (**Figure 3A**). Following intracytoplasmic staining, the gating strategy (**Figure 3B**, panel b) revealed that, in DNFB-treated mice, both CD4 (R1, **Figures 3B, a, c**) and CD8 (R2, **Figure 3C, a, b**) lymphocytes produced IL-13, and both antagonists inhibited its production. Notably, the inhibition effect was more pronounced in CD8^+^ lymphocytes. The repeated application of haptens to induce dermatitis usually triggers different effector profiles, inducing both type 1 and type 2 responses (29). When we evaluated this, we found that IFN-γ was not modulated by JNJ in either CD4^+^ lymphocytes (Th1) or CD8^+^ lymphocytes (**Figures 4A, B, a, b**). In contrast, in the presence of thioperamide, IFN-γ was significantly increased in CD4^+^ lymphocytes (**Figures 4A, a, b**).

Attending to the activation of T cells observed in the lymph node, we evaluated the recruited leukocytes in the ear. As shown in **Figure 4C**, we found that topical application of DNFB significantly increased the proportion of CD3^+^ cells, an effect lost in the presence of both antagonists. The decrease in T lymphocytes at the administration site could be associated with differential production of inflammatory cytokines and/or chemokines, indicative of less severe symptomatology. However, RT-PCR analysis did not enable us to find differences at the local level between treatments (**Supplementary Figure S1**). The results indicate that HA triggers a Th2/Tc2 profile in the DNFB allergy dermatitis model by acting on H_3_R and H_4_R. This is supported by the finding that the damage observed in the ear is notably reversed by the use of both antagonists, an effect that could be related to reduced recruitment of T cells and reduced edema and inflammation.

### Mechanisms associated with the inhibition of inflammation by the use of antagonists

**Figure 5:**
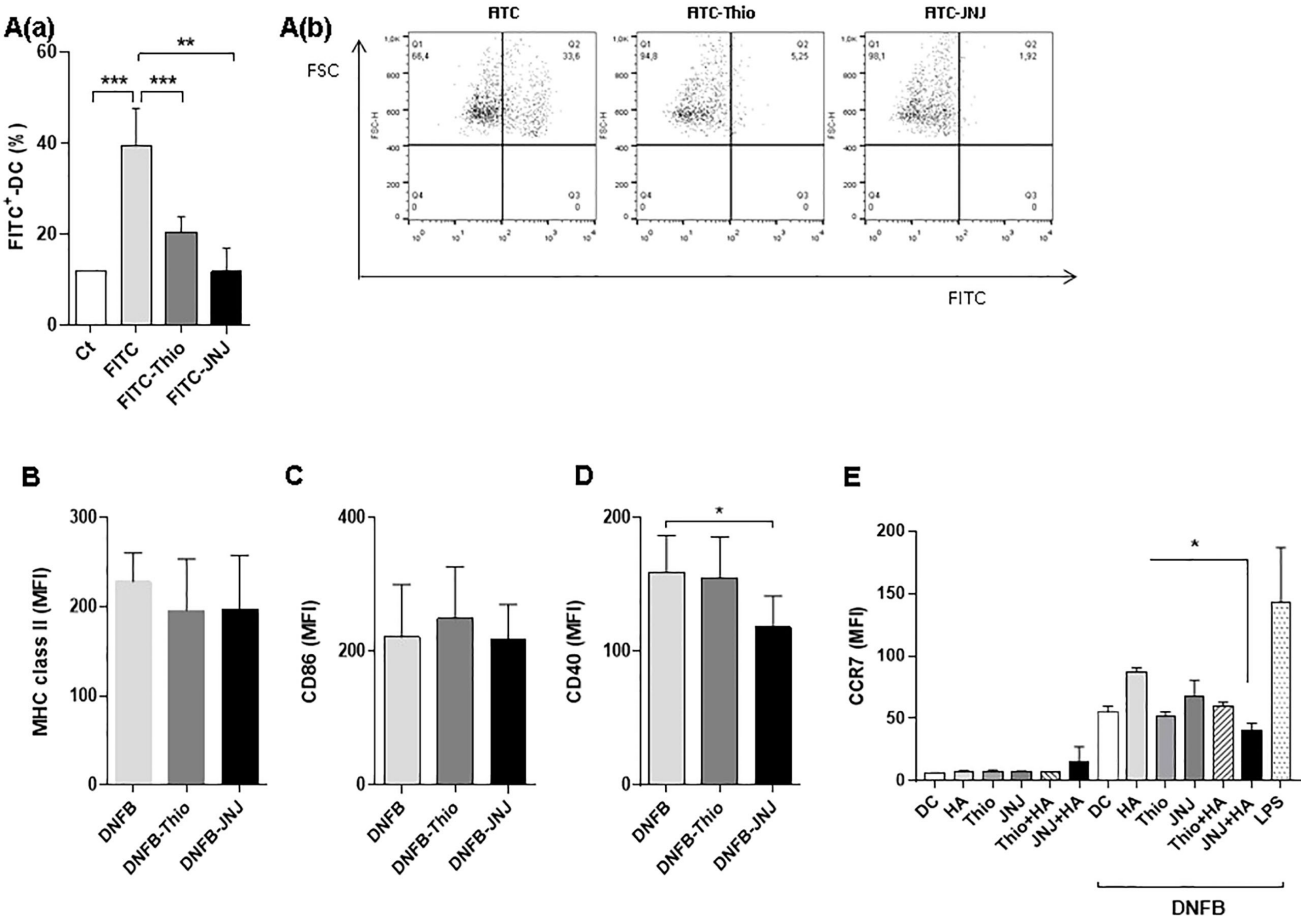
Analysis of migration of CD11c^+^IAd^+^ dendritic cells (DCs). Mice were treated topically with a 1% FITC solution with or without different inhibitors. The mean ± SEM FITC-labeled DCs recovered from zonal lymph nodes after 24 hours of application are shown (**A**, panel a). **p > 0.01,***p < 0.001. ANOVA with Tukey’s post-test (*n* = 4). Representative dot plots of each treatment are shown (**A**, panel b). The expression of MHC class II molecules (IA^d^; **B**), CD86 **(C)**, and CD40 **(D)** in DCs purified from mice treated topically with DNFB is shown. **(E)** Expression of CCR7 in DCs after 24 hours of stimulation *in vitro* (100 µg/ml DNFB). Bars represent the mean fluorescence intensity (MFI). *p < 0.05. ANOVA with Tukey’s post-test (*n* = 6).

To assess the cause of inhibition of inflammation, we evaluate the migration of DCs from the administration site to the lymph node. For this, we used FITC as a sensitizing hapten. FITC has similar characteristics to DNFB and is endocytosed by tissue DCs, inducing their migration to the lymph nodes. As seen in **Figure 5A**, the proportion of FITC^+^CD11c^+^IAd^+^ cells (DCs) in FITC-treated mice was significantly reduced after 6 hours in the presence of both antagonists. This reduced migration of DCs can in part be explained by an immature phenotype. When analyzing purified DCs, we found significantly reduced expression of the CD40 costimulatory molecule in the presence of the JNJ antagonist, whereas CD86 and MHC class II were not modified (**Figures 5B–D**). Even more interesting is the finding that H_4_R blockade markedly inhibits the expression of the CCR7 in DCs stimulated with DNFB *in vitro* (**Figure 5E**). This explains the lower migration to the lymph node, which, when added to the reduced expression of CD40 in DCs following H_4_R blockade, could explain the diminished activation of effector response and concomitant reduction in recruitment of CD3^+^ lymphocytes in the ear.

**Figure 6:**
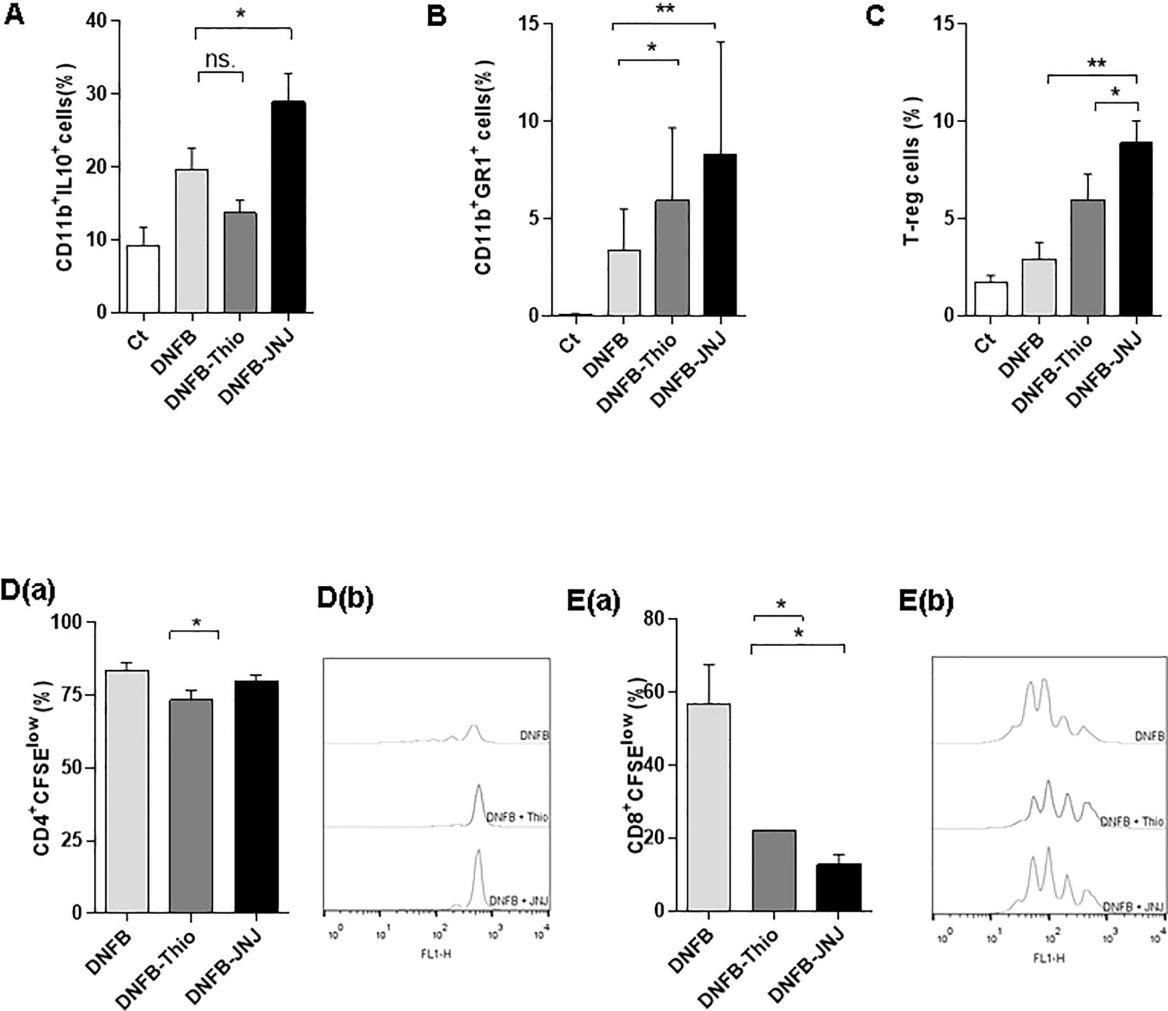
Induction of a regulatory phenotype in the presence of HA antagonists. **(A)** Intracytoplasmic staining of mononuclear cells stimulated *in vitro* for 24 hours with PMA (10 ng/ml) + ionomycin (1 ng/ml). Cells were treated with brefeldin for 6 hours, permeabilized, and then IL-10 was labeled and the cells were counted in a cytometer. Evaluation of proportion of myeloid cells **(B)** and regulatory T cells in permeabilized cells **(C)** from draining lymph nodes. (**D, E**, panel a) The determination of proliferation by dilution of CFSE is shown. Lymph node cells were labeled with the fluorescent dye CFSE and incubated for 96 hours in the presence of anti-CD3 (0.1 μg/ml) that was applied to the plate. A representative dot plot shows CD4 (**D**, panel b) and CD8 lymphocytes (**E**, panel b). Bars represent the mean ± SEM. ns (not significant); *p < 0.05; **p < 0.01. ANOVA with Tukey’s post-test (*n* = 9).

The induction of a tolerogenic environment would explain the decrease in effector cells in the tissue, as well as the inhibition of IL-13 production by CD4^+^ and CD8^+^ lymphocytes (**Figure 4**). As shown in **Figure 6A**, we found increased production of IL-10 in CD11b^+^ and CD11c^+^ cells in mice treated topically with JNJ. In addition, in mice treated with both antagonists, an increase in the proportion of CD11b^+^Gr1^+^ leukocytes was observed (**Figure 6B**), a characteristic phenotype of myeloid suppressor cells that are also associated with immunosuppression of the immune response (30). Furthermore, we found a significant increase in regulatory T lymphocytes (CD4^+^FOXp3^+^CD25^+^) when mice were treated with JNJ (**Figure 6C**). Together, the tolerogenic environment and the decreased migration of DCs to the draining lymph node may be associated with a decrease in effector response as a result of inhibition of the activation of naive T lymphocytes. To analyze this further, the proliferative capacity of T cells was evaluated using the CFSE cell tracer. As shown in **Figure 6**, the proliferation of both CD4^+^ T cells (**Figures 6D, a, b**) and CD8^+^ T cells (**Figures 6E, a, b**) is inhibited by the application of thioperamide, with a more pronounced effect in the CD8^+^ population. In contrast, the use of JNJ affected the proliferation of only CD8^+^ T lymphocytes.

**Figure 7:**
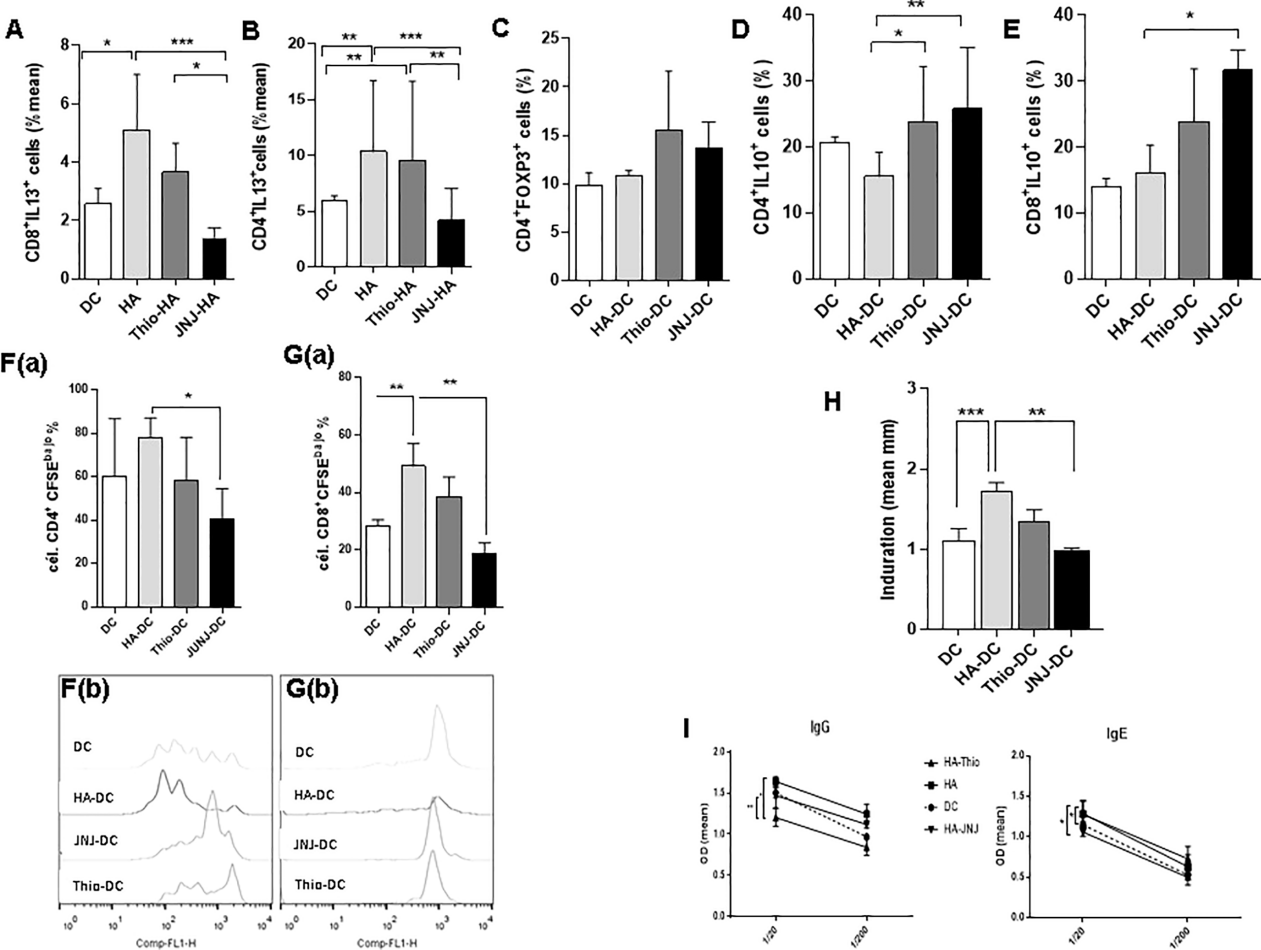
Blockade of signaling by blocking the H_4_R on DCs results in a tolerogenic profile. **(A, B)** The intracytoplasmic content of IL-13 is shown. Lymphocytes from draining lymph nodes were cultured for 24 hours with PMA (10 ng/ml) + ionomycin (1 ng/ml), with the addition of brefeldin A (10 µg/ml). Finally, cells were permeabilized and stained **(C)** FOXp3^+^CD4^+^ regulatory T cells stained from the draining lymph nodes of treated mice. Cells were permeabilized, then stained with the corresponding antibodies and analyzed by cytometry (*n* = 6). **(D, E)** IL-10 intracytoplasmic determination in lymphocytes cultured for 18 hours with PMA (10 ng/ml) + ionomycin (1 ng/ml), with the addition of brefeldin (10 µg/ml). Finally, cells were permeabilized, labeled, and analyzed by cytometry (*n* = 6). (**F, G**, panel a) Proliferation of leukocytes from the lymph node is shown. Cells were labeled with the fluorescent dye CFSE and incubated in plates pretreated with anti-CD3 (0.1 µg/ml). Ninety-six hours later, cells were analyzed by cytometry (*n* = 3). A representative experimental histogram is shown for CD4 and CD8 cells (**F, G**, panel b). **(H)** Induration was measured after 9 days of DC treatment. **(I)** Quantification of allergen-specific antibodies measured in serum of treated mice (*n* = 7). Bars represent the mean ± SEM. *p < 0.05; **p > 0.01; ***p < 0.001. ANOVA with Tukey’s post-test.

Next, we analyzed whether the modulation of the inflammatory response induced by HA results from its interaction with receptors expressed on DCs. For this, we developed the dermatitis model with a slight variation. We generated bone marrow DCs (BM-DCs) and treated them with the antagonists both *in vitro* and *in vivo*, by subcutaneously inoculating the antagonists into mice previously treated with topical DNFB. Analysis of the phenotype after treatment with DNFB *in vitro* showed that the blockade of the H_4_R decreases the expression of the CD40 and CCR7 molecules and that H_4_R antagonists also decrease the migratory capacity of BM-DCs (**Supplementary Figure S2**). After the analysis of the effector response induced, we found a decrease in IL-13 production in CD4^+^ and CD8^+^ lymphocytes from mice treated with BM-DCs stimulated with JNJ, but not in mice treated with thioperamide (**Figures 7A, B**). This finding could indicate that, although H_4_R is expressed mainly in leukocytes, H_3_R is expressed at the level of the nerve endings of the CNS, lung, and skin (12), and so the effect observed by topical application to the ear would be lost when blocking H_3_R in DCs with thioperamide.

Finally, we analyzed the mechanisms associated with the inhibition. Although we did not find differences in regulatory T cells between treatments (**Figure 7C**), we did find an increase in IL-10 production in CD4^+^ and CD8^+^ T cells (**Figure 7D**). As shown in **Figure 7**, the proliferation of CD4 (**Figure 7F**, panels a and b) and CD8 (**Figure 7G**, panels a and b) lymphocytes was inhibited in the presence of JNJ, and, although thioperamide appears to have an effect in both populations, it is not significant. These results lead us to infer that the effector response induced by the DNFB is blocked by inducing an immunosuppressive profile. This environment is generated thanks to the functional blockade of H_3_ and H_4_ histaminergic receptors, which triggers a regulatory T-cell response and the production of the anti-inflammatory cytokine IL-10. Most importantly, we have shown that immunosuppression is associated with an improvement in allergic symptoms, as evidenced by reduced inflammation of the ear after treatment with DC-JNJ (**Figure 7H**) and specific IgE antibodies (**Figure 7I**).

## Discussion

We previously demonstrated that HA increases the recruitment of an unconventional profile of CD8 lymphocytes in the lungs of mice allergic to ovalbumin (31). Interestingly, when interacting with murine DCs, HA enhances the cross-presentation of antigens through the vacuolar pathway (32). Non-conventional CD8 lymphocytes of the Tc2 and/or Tc17 type have been described in allergic patients (8), a profile that is associated with the potentiation of the inflammatory response and the chronicity of these pathologies (3). The aim of this work was to deepen our understanding of the mechanisms associated with the induction of unconventional responses by HA in allergic CD. To do this, we developed a model of allergic dermatitis by repeated application of DNFB, as other studies have shown that topical application of DNFB increases serum levels of HA, with reported values ranging from 20 to 40 ng/ml, and also increases mast cell recruitment in local tissue (33–35). We have shown that DNFB increases degranulated mast cells in the ear, resulting in the release of mediators, including HA. The topical application of antagonists inhibited this effect, resulting in a reduced inflammatory response. We also found that the application of H_4_R antagonists reverses the inflammatory effect associated with the Th2/Tc2 profile, a mechanism that involves an immunosuppressive response, dependent, in part, on IL-10 production, leading to an improvement in the symptoms associated with dermatitis. Related to this, a high proportion of non-conventional CD8^+^ T cells specific for antigen 1 (Derp-1) was demonstrated in individuals with atopic dermatitis caused by *Dermatophagoides pteronyssinus* (36). Although the presence of these lymphocytes correlates with the severity of allergic diseases, the mechanisms through which this unconventional response profile is induced are still unknown. However, it is known that depletion of CD8^+^ T cells prevents airway hyperresponsiveness (37). Similarly, CD8^+^ T lymphocytes, which produce the specific allergens IL-13 and IL-5, have been isolated in high numbers from skin lesions due to allergic reactions (36).

It should be noted that atopic patients refractory to corticosteroids have a low capacity to produce IL-10 (38), and this is linked with the severity of the pathology. It has been shown that CD8 Tc2 lymphocytes are more resistant to corticosteroids than CD4 lymphocytes (39), and this finding can be explained, in part, by reduced expression of the ATF2 protein necessary for the transactivation and production of IL-10 in response to steroids (40). This explains, in part, why the increase in the percentages of unconventional CD8 lymphocytes in asthma and dermatitis correlates with the severity of the disease (36, 38). Therefore, it is relevant in our model that dual blockade of H_3_R/H_4_R or blockade of H_4_R specifically induces a suppressive microenvironment associated with the increment of FOXp3^+^ regulatory T lymphocytes. Previous work has shown that, in patients with atopic dermatitis, the prevalence of regulatory CD8^+^ T lymphocytes in lesions is low, although there is an increase in the number of CD8^+^ Tc2 lymphocytes in the peripheral blood (41, 42). In contrast, other studies have found increased recruitment of regulatory T cells in dermatitis lesions, although it is thought that these T cells do not play an efficient immunosuppressive role (43, 44). In our model of dermatitis, we found that the HA receptor antagonists thioperamide and JNJ suppress the type 2 response (Th2/Tc2), which is associated with a higher proportion of FOXp3^+^ lymphocytes with functional activity, as we found inhibition of T-cell proliferation. Strikingly, the specific H_4_R antagonist induced an increase in myeloid suppressor cells (CD11b^+^GR1^+^); however, we cannot rule out the possibility that these cells may also have functional relevance in the containment of the inflammatory response, for example by suppressing T-cell proliferation, nitric oxide production, metalloproteinase activity, or the production of inflammatory cytokines.

Since its discovery, the H_4_R has become an attractive therapeutic target not only for asthma, but also for other allergic diseases, such as dermatitis, allergic rhinoconjunctivitis, and psoriasis (16, 18, 45). We have shown that H_4_R antagonists inhibit the production of the Th2 chemokines CCL17 and CCL22 in human monocyte-derived Langerhans cells from patients with atopic dermatitis (46). Likewise, in mice deficient in H_4_R, fewer skin lesions were observed as a consequence of a lower activation of CD4^+^ lymphocytes and a reduction in specific IgE. Surprisingly, the use of JNJ28307474, an H_4_R antagonist, partially reversed the inflammatory effects when applied during the sensitization and challenge phase, but not when applied during the effector phase (17). In a TNCB CD model, Seike et al. (47) demonstrated that, when HA interacts with the H_4_R, it acts as a chemotactic agent for eosinophils and mast cells that access eczematous lesions.

Because monotherapies have limited and/or opposing effects in allergic diseases, the focus has switched to the combined use of H_4_R and H_1_R antagonists as palliatives for allergic dermatitis and ACD, and progress has been made recently. For example, Mashushita et al. (24) have demonstrated, in a mouse model of TNFB-induced CD, that IgE and the Th2 cytokines IL-4, IL-5, and IL-6 in eczematous lesions are reduced by combined therapy. In line with this, we found that the dual H_3_R/H_4_R antagonist thioperamide is more efficient than JNJ in reducing cutaneous lesions associated with tissue edema, probably, as previously shown (17, 48), because of their interaction with H_3_ at the level of histaminergic nerve endings in the skin is responsible for itching. It should be noted that, although H_3_R and H_4_R (12, 28) are highly homologous, and thioperamide interacts with both receptors, thioperamide interacts with H_3_R and H_4_R with different affinity, having a higher affinity for H_3_R, and this explains why some effects are similar with both antagonists and others are not. It should also be noted that JNJ7777120 was originally designated a selective antagonist receptor for human H_4_R, and was later shown to behave as a partial agonist in other H_4_R orthologous species (e.g., rats, mice, dogs) with respect to Gi protein activation and also as a partial agonist, recruiting β-arrestin, in humans (49, 50). Therefore, the concentration of HA determines its action: at low levels, it acts as an agonist. Throughout our work its actions antagonize the effects of the amine. In fact, to the best of our knowledge, for the first time in an allergic dermatitis model, we demonstrate that HA signaling *via* H_4_R expressed in DCs induces an inflammatory response associated with the activation of Th2 effector lymphocytes, and also non-conventional CD8 Tc2 lymphocytes that are associated with the severity of the pathology (38), as specific antibodies and effector recruitment are increased. Although several studies have demonstrated an effect of HA on DC migration *via* H_4_R, and this is due to an increase in CCL19/21 chemokines (46, 51), the expression of its ligand was not shown to be affected by HA. For example, Caron et al. (52) did not observe an alteration in the expression of the receptors for the chemokines CCR7, and CCR5, among others, in human immature DCs. In our model, immature DCs do not modify the expression of CCR7, but, strikingly, when treated with the hapten, HA, by interacting with the H_4_R, increases the expression of CCR7. This would explain the lower migration of DCs to the zonal lymph node in mice treated with topical DNFB and JNJ, which, when added to the immature phenotype and the production of IL-10, would stimulate a tolerogenic response. It should be noted that the blockade of H_3_R/H_4_R in DCs by the use of thioperamide did not show the same effect. We believe that this is related to the fact that the dual antagonist does not reach sufficient concentrations or have sufficient affinity to completely block H_4_ receptors on DCs. Neumann et al. (53), in a murine model of asthma, demonstrated that thioperamide is not comparable to JNJ because their pharmacokinetics are different. Neumann et al. (53) also observed that some effects were induced by both antagonists, whereas others, such as specific IgE inhibition and pulmonary infiltration, were not. We posit that the rational use of specific H_3_R/H_4_R antagonists could improve the symptoms associated with dermatitis.

## Conclusion

In light of the results obtained in this work, we believe that future experiments should consider the combined use of H_3_R and/H_4_R antagonists, which would be beneficial in reducing the symptoms associated with dermatitis, as blocking the H_3_R would decrease the scratching action, which is an initial event in these pathologies, inducing damage to the epithelium that triggers the initial inflammatory response, and blocking H_4_R would induce a tolerant microenvironment by interacting with tissue DCs.

## Data availability statement

The original contributions presented in the study are included in the article/**Supplementary Material**. Further inquiries can be directed to the corresponding author.

## Ethics statement

The animal study was reviewed and approved by Institutional Committee for the Care and use of Laboratory animals (CICUAL) and Biosafety committee.

## Author contributions

JA and MV designed the research. JA and AIC performed the research. SV and GB performed IQ and analysis. JA analyzed the data. GS and MV supervised the work and JA and MV wrote the paper. All authors contributed to the article and approved the submitted version.

## Funding

This research was supported in part by the PIP-2017 #0950 granted by the Consejo Nacional de Ciencia y Tecnología (CONICET) and PICT-SERIEA-2020-03322 granted by FONCYT from Argentina.

## Conflict of interest

The authors declare that the research was conducted in the absence of any commercial or financial relationships that could be construed as a potential conflict of interest.

## Publisher’s note

All claims expressed in this article are solely those of the authors and do not necessarily represent those of their affiliated organizations, or those of the publisher, the editors and the reviewers. Any product that may be evaluated in this article, or claim that may be made by its manufacturer, is not guaranteed or endorsed by the publisher.
