# Supplementary material for: Mechanisms of unconventional CD8 Tc2 lymphocyte induction in allergic contact dermatitis: Role of H3/H4 histamine receptors
Source: Front Immunol. 2022 Oct 7;13:999852. doi: 10.3389/fimmu.2022.999852 (PMC9586454; doi:10.3389/fimmu.2022.999852)
Supplement: Supplementary file 1 [file DataSheet_1.pdf]

Antibody	Clone	Reference	Source	Isotype
Anti-mouse-IA-d	39-46-8	553548	BD Pharmingen	Mouse IgG2b,κ
Anti-mouse-CD11c	HL3	553801	BD Pharmingen	Armeniam hamster IgG1,λ2
Anti-mouse-CD11b	M1-70	553310	BD Pharmingen	DA/HA IgG2b,κ
Anti-mouse-GR1	RB6-8C5	22155244	Immunotools	Rat IgG2b
Anti-mouse-CCR7	4B12	12-1971-82	eBiosciences	Rat IgG2a,κ
Anti-mouse-CD8	H35-17.2	550798	BD Pharmingen	Rat IgG2b,κ
Anti-mouse-CD4	RM4-5	561090	BD Pharmingen	Rat IgG2a,κ
Anti-mouse-Foxp3	FJK-16s	11-5773-82	ebiosciences	Rat IgG2a,κ
Anti-mouse-CD25	3C7	553075	BD Pharmingen	Lewis IgG2b,κ
Anti-mouse-CD40	HM40-3	553723	BD Pharmingen	Rat(Lou)IgG2a,κ
Anti-mouse-IL-13	EBio13A	12-7133-81	eBiosciences	IgG1,κ
Anti-mouse-IFNγ	XMG1.2	11-7311-81	eBiosciences	IgG1,κ

Table S1: Detail of the antibodies used in the experimental procedure

Name of oligonucleotide	Sequence Fw (5´to 3´)	Sequence Rv (5´ to 3´)	Specificity
IL-10	ACCAAAGCCACAAAGCAG	GCCAGTCAGTAAGAGCAGG	mouse
IL-17	TTTCAGCAAGGAATGTGGA	AACACGAAGCAGTTTGGG	mouse
IL-5	ATGAAGTGCTGGAGATGGAA	GCAACGAAGAGGATGAGGG	mouse
IL-13	TCTTGCTTGCCTTGGTGC	TGGTCTTGTGATGTTGCT	mouse
CCR7	TTAGCAAGCAAGCAAGAGATGA	CGGTGGAGAGGCAGAAGG	mouse
CXCR3	GTCCTCCTTGTAGTTGGGCT	TGGTGTTGTCCTTGTTGCTG	mouse

Table S2: Oligonucleotide sequences in Real-time PCR
