# Supplementary figures and images for: Mechanisms of unconventional CD8 Tc2 lymphocyte induction in allergic contact dermatitis: Role of H3/H4 histamine receptors
Source: Front Immunol. 2022 Oct 7;13:999852. doi: 10.3389/fimmu.2022.999852 (PMC9586454; doi:10.3389/fimmu.2022.999852)

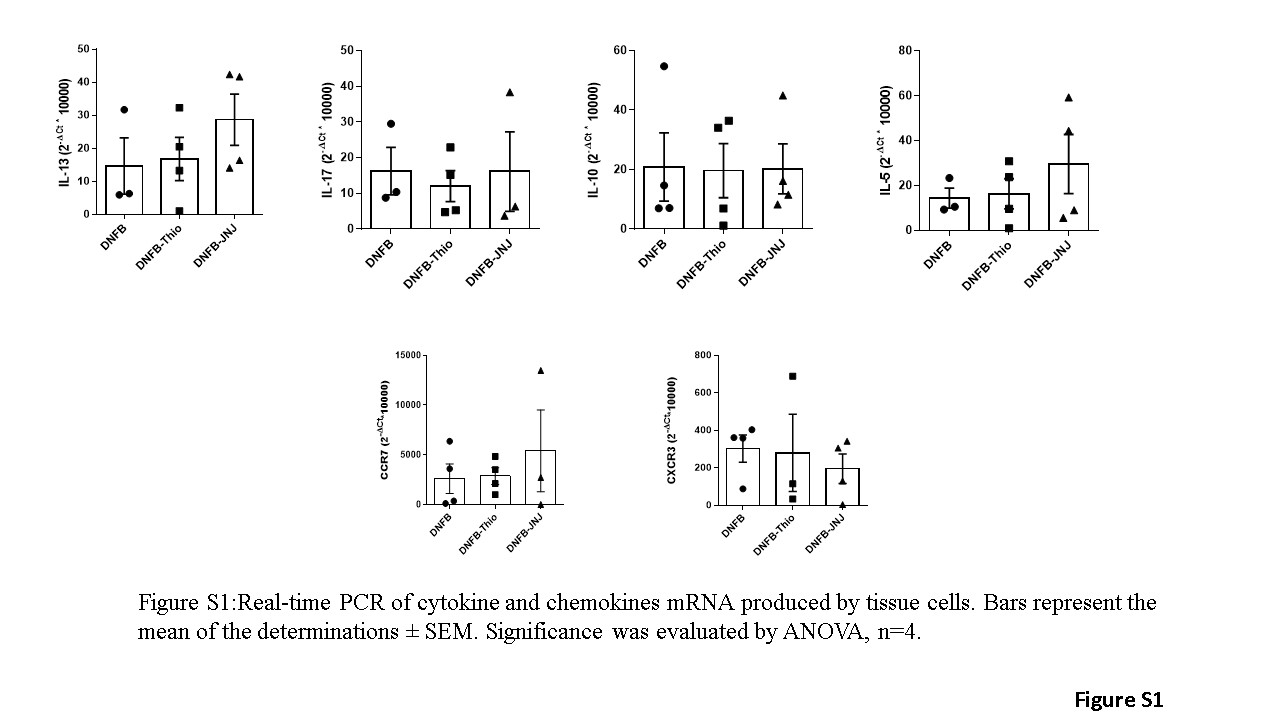

Supplement: Supplementary file 2 [file Image_1.jpeg]

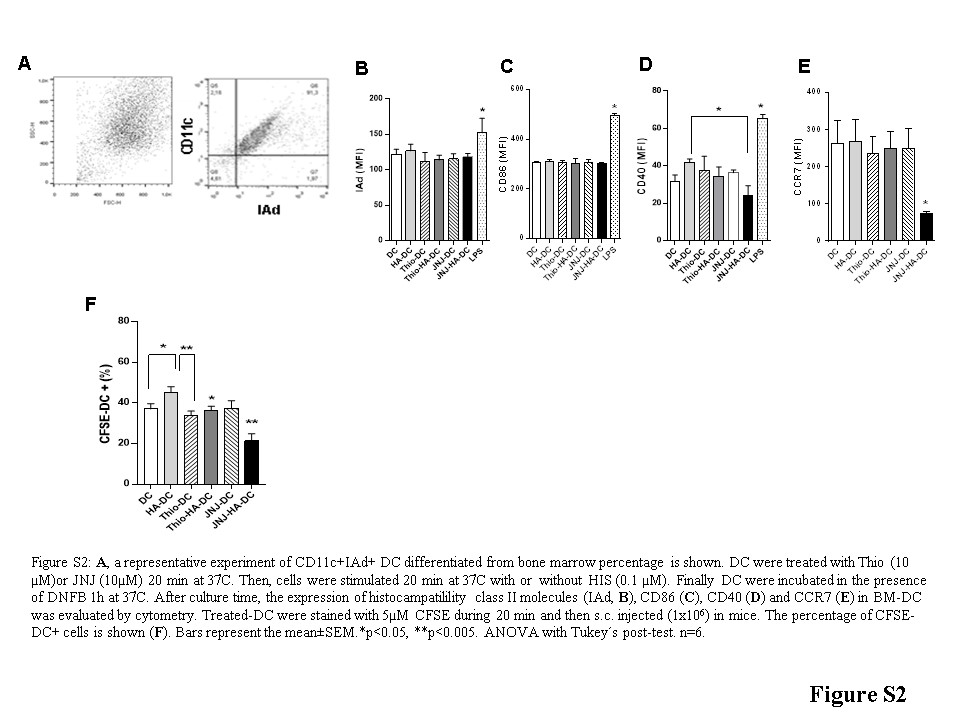

Supplement: Supplementary file 3 [file Image_2.jpeg]
